# Different Contexts in the Oddball Paradigm Induce Distinct Brain Networks in Generating the P300

**DOI:** 10.3389/fnhum.2018.00520

**Published:** 2019-01-07

**Authors:** Fali Li, Chanlin Yi, Yuanling Jiang, Yuanyuan Liao, Yajing Si, Jing Dai, Dezhong Yao, Yangsong Zhang, Peng Xu

**Affiliations:** ^1^MOE Key Lab for Neuroinformation, The Clinical Hospital of Chengdu Brain Science Institute, University of Electronic Science and Technology of China, Chengdu, China; ^2^Center for Information in Medicine, School of Life Science and Technology, University of Electronic Science and Technology of China, Chengdu, China; ^3^School of Computer Science and Technology, Southwest University of Science and Technology, Mianyang, China

**Keywords:** background context, brain network, P300, response variances, oddball paradigm

## Abstract

Despite the P300 event-related potential (ERP) differences between distinct stimulus sequences, the effect of stimulus sequence on the brain network is still left unveiled. To uncover the corresponding effect of stimulus sequence, we thus investigated the differences of functional brain networks, when a target (T) or standard (S) stimulus was presented preceding another T as background context. Results of this study demonstrated that, when an S was first presented preceding a T (i.e., ST sequence), the P300 experiencing large amplitude was evoked by the T, along with strong network architecture. In contrast, if a T was presented in advance [i.e., target-to-target (TT) sequence], decreased P300 amplitude and attenuated network efficiency were demonstrated. Additionally, decreased activations in regions, such as inferior frontal gyrus and superior frontal gyrus were also revealed in TT sequence. Particularly, the effect of stimulus sequence on P300 network could be quantitatively measured by brain network properties, the increase in network efficiency corresponded to large P300 amplitude evoked in P300 task.

## Introduction

In the oddball paradigm, two types of stimuli, i.e., target (T) and standard (S), are randomly presented to subjects, the presentation of T can evoke a P300 event-related potential (ERP; Squires et al., 1975; Duncan-Johnson and Donchin, 1977). To evoke a clear P300, subjects are required to only respond to T in a requested manner (e.g., counting the number or pressing a button) while omitting S in the active paradigm; while in the passive paradigm, subjects would only concentrate on stimuli without responses (Pokorny et al., 2013; Risetti et al., 2013). The P300 shows a largest positive peak at approximately 300 ms after stimulus onsets, and is found prominently over parietal region (Polich, 2007). The P300 can be used as a physiological biomarker to evaluate brain potential of information processing (Rutiku et al., 2015; Turetsky et al., 2015), and is widely used in clinical diagnosis (Howe et al., 2014; Turetsky et al., 2015; Li et al., 2018b), cognitive neuroscience (van Dinteren et al., 2014; Wang et al., 2015), and brain-computer interface (BCI; Farwell and Donchin, 1988; Yin et al., 2016). A hybrid BCI system combining P300 and steady-state visual evoked potential acquired an online classification accuracy of 93.85% with information transfer rate of 56.44 bit/min in only a single trial (Yin et al., 2013), which also achieved the wheelchair control (Li et al., 2013) and detection of awareness in patients with disorders of consciousness (Pan et al., 2014).

The effect of stimulus sequence on P300 has been investigated since 1976 (Squires et al., 1976; Verleger, 1987). A large P300 amplitude can be evoked, if identical stimuli (e.g., standard) are consecutively presented and then terminate with a different stimulus (e.g., target; Squires et al., 1976); an increase of the number of S stimulus before a T corresponds to the increase of P300 amplitude evoked by the T stimulus (Polich and Bondurant, 1997). Meanwhile, stimulus sequence interacts with interstimulus interval (ISI) and target probability. Small target probability produces a sequence that includes a series of S stimuli and a T stimulus, which evokes a clear P300. In contrast, the relatively short ISI consumes more brain resources, due to the frequent presentations of stimulus events, small P300 amplitude will be evoked (Polich, 1990).

Particularly, manipulation of global (especially local) T probability produces the effect of target-to-target interval (TTI) on P300. TTI determines how quickly brain resources can be redirected to process target information (Pashler, 1994); shorter interval produces much smaller P300 than longer interval (Steiner et al., 2013). The positive relationships between TTI and hemodynamic activity in multiple brain regions, such as anterior cingulate, might index that TTI modulates the brain activity related to target in distinct network structures by updating of working memory processes (Stevens et al., 2005).

Time-frequency analysis is of great importance to assess brain activity in ERPs (Busch et al., 2006; Bernat et al., 2007). The generation of P300 is attributed to a series of procedures that include stimuli perception, information integrating, decision processing, and neuronal response (Li et al., 2016). In fact, P300 activity varies in time-frequency amplitude and topography (Demiralp et al., 2001; Friedman et al., 2001). Brain activity in delta and theta bands is demonstrated to underlie P300 ERP (Başar-Eroglu and Demiralp, 2001; Harper et al., 2014; Bender et al., 2015). In order to investigate the change of brain activity during P300 task, the time-frequency analysis can be used (Friedman et al., 2001; Bernat et al., 2007).

In the brain, information is processed between specialized and spatially distributed but functionally linked regions (Bullmore and Sporns, 2009; Tian et al., 2013; Zhang et al., 2015; Bassett and Sporns, 2017; Li et al., 2018a). The endogenous processing in the brain, which functions on large-scale areas including frontal and parietal lobes (Bledowski et al., 2004; Polich, 2007) and their interactions (Zhang et al., 2014; Li et al., 2015, 2016), contributes to the generation of P300 (Donchin and Coles, 1988). Lesions of brain regions, such as temporal-parietal junction, resulted in P300 deficits, i.e., the decreased amplitude and prolonged latency (Yamaguchi and Knight, 1991; Verleger et al., 1994).

There is a debate that which aspect of cognitive process in the brain can be reflected by the P300 (Linden, 2005; Polich, 2007; Rutiku et al., 2015), decision (O’Connell et al., 2012), stimulus-response links (Verleger et al., 2014), or attentional resources allocation (Putnam and Roth, 1990). In order to investigate this debate, the effects of stimulus characteristics on P300 were studied (Polich and Bondurant, 1997; Steiner et al., 2013). Despite the differences of P300 characteristics (e.g., amplitude and latency), no findings on brain networks were reported for this issue. In this study, we assumed that when distinct contexts were first presented, the brain network architectures with varied efficiencies were activated, and then contributed to the generation of P300. Taking a T as the reference, two types of stimulus sequences exist; one sequence is an S preceding a T (ST sequence), and another occurs when a T is presented in advance (TT sequence). We then investigated how the brain adapted dynamically to external stimuli, and probed the effects of different contexts on P300 by comparing the brain networks between different stimulus conditions.

## Materials and Methods

### Participants

This experiment was approved by the Institution Research Ethics Board of University of Electronic Science and Technology of China. Twenty-two healthy graduate students (males, age of 22–27 years) were compensated financially to participate in P300 experiments, after providing their signed written informed consent. All participants were right-handed (self-reported), and had the normal or correct-to-normal vision. None of them had a history of substance abuse and a personal or family history of psychiatric or neurological disease.

### Experiment Design

**Figure 1:**
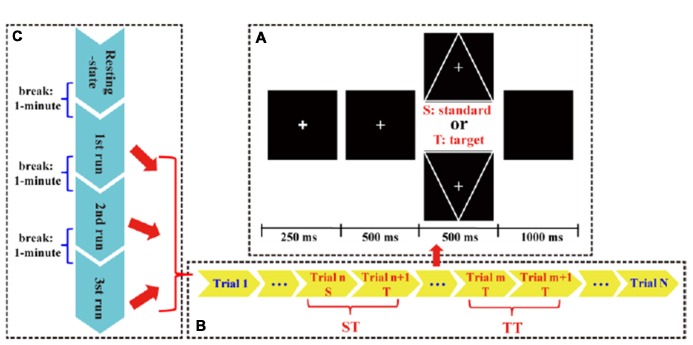
The P300 protocol used in this study. **(A)** Each trial consisted of an alert of attention (250 ms), a cue of preparation (500 ms), a target (T) or standard (S) stimulus (500 ms), and a short black screen (1,000 ms). **(B)** Each run consisted of 150 S/T stimuli. **(C)** Whole P300 task consisted of three runs.

In this study, the T and S stimulus were expressed as a downward- and upward-oriented triangle with a thin cross in the center, respectively. For each stimulus, the edge length of the triangle was 4° visual angle. The black color was set as background color, and the white color was set as triangle color. The line widths in triangles were 1 mm, and the triangles were isosceles. Before task, all subjects were required to be relaxed, to focus attention on task without extensive body motion, and to avoid eyes blinking. Details of experimental protocols (Figure 1) were depicted as follows.

In this study, an eyes-closed resting-state with a duration of 4 min and three runs of P300 tasks were included. Between each two adjacency periods, a short break (i.e., 1 min) was given to subjects. Each run consisted of 150 trials (120 standards and 30 targets) that were randomly presented. In each trial, a bold solid cross appeared as an alert to warn subjects to focus their attention; after 250 ms, a thin solid cross appeared as a cue to inform subjects that either a T or S would appear very soon; after 500 ms, a stimulus was presented for 500 ms, and at the same time, subjects were required to count the number of T stimulus. After a break with the 1 s duration, next trial initiated. When each run ended, subjects would verbally speak out the counted number.

### Data Recording

Rest and task electroencephalogram (EEG) data sets of 64 Ag/AgCl electrodes that were positioned according to international 10/20 system were recorded by using BrainVision 2.0.1 (Brain Products, Munich, Germany). The predefined parameters of online bandpass filtering was 0.01–100 Hz, and sampling rate was 500 Hz. During online recording, electrodes FCz and AFz were regarded as reference and ground, respectively. Two additional electrodes, i.e., vertical (VEOG) and horizontal electrooculograms (HEOG), were used to monitor the eye movements. Particularly, VEOG was positioned at the right side of right eye, and HEOG was positioned below the left eye. During whole task, impedances for all electrodes were kept below 5 KΩ.

### Data Analyses

In this study, three runs of task data sets were preprocessed with preprocessing procedures including, offline detecting and correcting blinks by using BrainAnalyzer 2.0.1, re-referencing to a neutral reference of Reference Electrode Standardization Technique (REST; Yao, 2001; Dong et al., 2017), 0.5–30 Hz bandpass filtering, 4.5 s data segmenting, and artifact-trial removal (±75 μv as threshold). Particularly, when performing data segmentation, we extracted two adjacent trials with the 2.25 s duration per trial, i.e., an S and a T of ST sequence and two T of TT sequence. Meanwhile, for artifact-trial removal, the 75 μv threshold was used to automatically exclude artifacts with high amplitudes. If trial with largest absolute value of amplitude at any time point of any electrode exceeded 75 μv, this trial would be excluded from any further analysis. In this study, only two subjects experienced an exclusion of large trial number (ST: 11 and 16 trials, and TT: 6 and 5 trials). All trials (17.08 ± 3.30) in TT sequence were used in our analysis. Meanwhile, to acquire reliable result and to exclude the effect of trial number, based on the trial range of TT sequence, for ST sequence, we randomly selected the trial number within the same range for each subject, and 17.52 ± 1.75 trials in ST sequence were thereby included in our analysis. Thereafter, the trial numbers were then statistically investigated between two sequences by performing paired *t*-test, and no significant difference (*p* = 0.579) between two sequences was found.

#### Time-Frequency Analysis

Brain activity in low frequency range (i.e., delta and theta) is found to relate to the P300 (Harper et al., 2014; Bender et al., 2015). In this study, aiming to investigate the dynamic of task-related brain activity in frequency domain, we acquired the time-frequency distributions (TFDs) for two sequences by using the wavemenu of Matlab (v2014a). Particularly, we first calculated the TFDs for each artifact-free ST (also for TT) segment, and the TFDs were then averaged across segments to achieve TFDs mean of ST (also TT) sequence for each subject. Afterwards, to clearly illustrate the dynamic of brain activity, the TFDs mean was also grand-averaged across subjects.

#### P300 Measurements

One second interval of [−200, 800] ms (0 ms represents the T onsets) was used to estimate the P300 amplitude. In this study, we averaged the T segments to achieve an averaged P300 ERP per condition for each subject. Afterwards, within time interval of [300 ms, 600 ms] after T onsets, P300 amplitude on electrode Pz was calculated by averaging amplitudes within a time window of ±10 ms centered at largest positive peak. Meanwhile, we also estimated the corresponding P300 latency by obtaining the time point corresponding to the largest positive peak.

#### Brain Networks

The brain network is typically modeled by graph theory, and includes a collection of nodes and edges. In this study, 21 electrodes were set as network nodes, and the synchronized strengths between electrodes estimated by coherence were set as network edges. Coherence is usually used to measure the synchronized neuronal assembly at any given frequency bin *f* between two signals, *x*(*t*) and *y*(*t*), and is formulated as,

(1) $$C_{xy}(f)={\left|R_{xy}(f)\right|}^{2}=\frac{{\left|P_{xy}(f)\right|}^{2}}{P_{xx}(f)P_{yy}(f)}$$

where *C_xy_*(*f*) and *R_xy_*(*f*) represent the coherence value and complex correlation coefficient between *x*(*t*) and *y*(*t*) at frequency *f*, respectively. *P_xy_*(*f*) represents the cross-spectrum of *x*(*t*) and *y*(*t*) at frequency *f*, and *P_xx_*(*f*) and* P_yy_*(*f*) represent the auto-spectrum at frequency *f* of *x*(*t*) and *y*(*t*), respectively. These measurements of spectral densities were calculated from Fast Fourier Transform. For each frequency bin *f*, the *C_xy_*(*f*) is acquired by squaring the magnitude of the complex correlation coefficient *R*, which returns a real value within the range of [0, 1].

The short time series cannot exactly estimate the correlations between two electrodes, and may result in the spurious estimation of P300 network. In this study, EEG segments with relatively long interval (Chen et al., 2014; Diez et al., 2017), 2.25 s duration, were used to construct P300 networks. Meanwhile, P300 networks would be constructed in low frequency range, i.e., 1–10 Hz of our present study.

In this study, based on these artifact-free EEG segments, the coherence was first used to estimate the synchronized strength, *C_xy_*(*f*), between two electrodes at frequency *f* per segment. Then, we averaged the synchronized strengths within the interested frequency range of 1–10 Hz to form an adjacency matrix per segment for each subject. Afterwards, for each subject, the final weighted EEG network that corresponded to either S or T of either ST or TT sequence, an adjacency matrix with dimension of 21 × 21, was obtained by averaging adjacency matrices across all artifact-free segments for each condition.

Network properties including clustering coefficients (CC) and characteristic path length (CPL) can be used to quantitatively measure brain network (Rubinov and Sporns, 2010). Theoretically, the CC indexes the functional segregation of a given network, and reflects the capacity for specialized processing to occur within densely interconnected regions. In contrast, the CPL is usually used to measure the corresponding functional integration, and indexes the ability to rapidly combine specialized information from distributed brain regions. In this study, we used the brain connectivity toolbox (BCT^1^) to calculate the CC and CPL. Here, *w_ij_* represents the synchronized strength between nodes *i* and *j* estimated by coherence, *d_ij_* represents the shortest weighted path length, *N* represents the number of all nodes, and Θ represents the set of network nodes. The CC and CPL were then formulized as follows:

(2) $$CC=\frac{1}{N}\sum_{i\in \Theta }\frac{\sum_{j,l\in \Theta }{\left(w_{ij}w_{il}w_{jl}\right)}^{1/3}}{\sum_{j\in \Theta }w_{ij}\left(\sum_{j\in \Theta }w_{ij}-1\right)}$$

(3) $$CPL=\frac{1}{N}\sum_{i\in \Theta }\frac{\sum_{j\in \Theta ,j\neq i}d_{ij}}{N-1}$$

#### Statistical Analysis

To investigate how the brain adjusted from last stimulus to adapt to a T stimulus, we then compared the EEG networks between two adjacency stimuli (i.e., 22 × 21 × 21 of stimulus 1 vs. 22 × 21 × 21 of stimulus 2) in both sequences by using paired *t*-test, which resulted in a 21 × 21 matrix whose elements denoted significant (*p* < 0.05) or insignificant (*p* > 0.05) differences between two conditions. Particularly, if a network edge experienced significantly different between two conditions, this edge would be given an exact value (i.e., the quantitative difference of edge strengths between two stimuli); in contrast, if no significant difference was found, this edge would be discharged (i.e., giving a zero value). Thereafter, the 21 × 21 matrix was drawn on the scalp to visually display the topological differences between two stimuli, by using BCT function (i.e., “Brain_Graphic”).

Afterwards, the paired *t*-test was also used to compare the P300 amplitude/latency and network properties between two stimuli to investigate the effect of stimulus sequence, as well as the differences of task activations between T in ST and T2 in TT. In this study, all comparisons were multiple corrected by performing false discovery rate (FDR) test.

#### Differential Cortical Sources

To investigate the brain regions that were important when responding to the second T in both sequences, we first estimated the standardized current densities that corresponded to trial-averaged ST/TT ERPs, by using standardized low-resolution electromagnetic tomography (sLORETA; Pascual-Marqui, 2002). The mean of sLORETA solutions within a time window of ±10 ms centered at their own largest positive peak within 300–600 ms after stimulus onsets was then calculated and compared between two sequences. Considering that deep brain regions, such as parahippocampal gyrus, contributed little to scalp signals, and were demonstrated to have weak activations. In this study, these regions were also excluded from any further analysis.

In this study, the sLORETA employed the current density estimate given by minimum norm solution, and was capable of exact (zero-error) localization. In sLORETA, the solution space consists of 6,239 voxels (also includes hippocampi) at 5 mm spatial resolution in a 3-shell realistic head model (Fuchs et al., 2002), using the digitized Montreal Neurological Institute atlas (152 template; Mazziotta et al., 2001).

## Results

**Figure 2:**
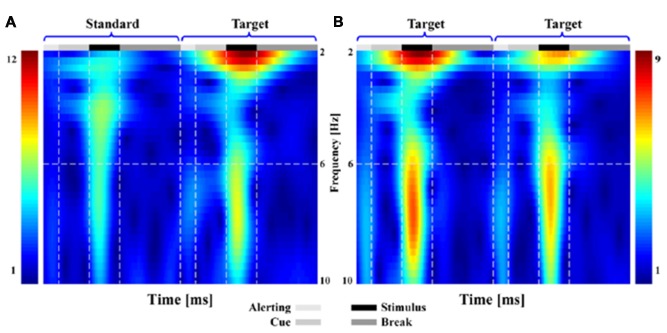
The time-frequency distributions (TFDs) related to brain activity for both standard-target (ST) and target-to-target (TT) sequences during P300 tasks. Subfigure **(A,B)** denote the TFDs of ST and of TT on electrode Pz, respectively.

**Figure 3:**
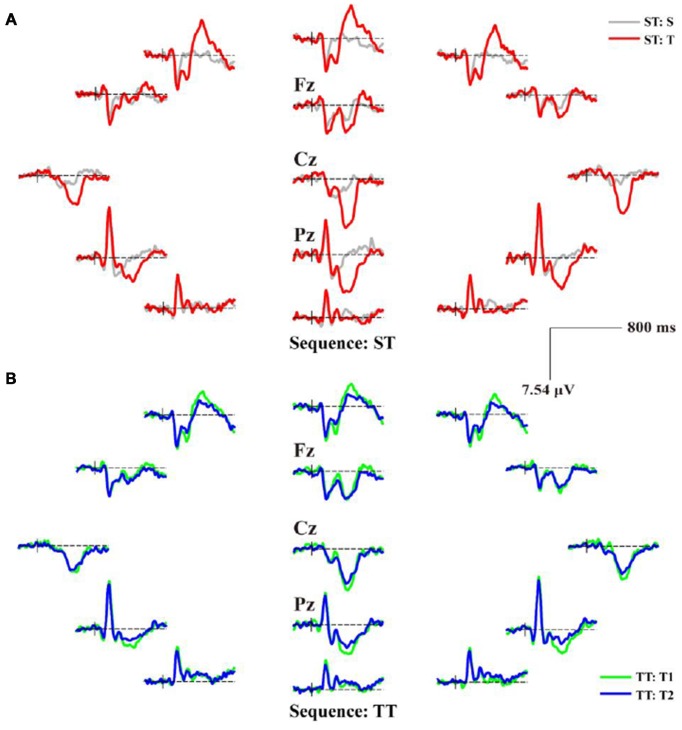
The scalp distributions of averaged P300 event-related potentials (ERPs) for ST and TT sequences during P300 task. **(A)** P300 ERPs of ST. **(B)** P300 ERPs of TT. In **(A)**, the gray and red solid lines denote the waveforms evoked by the S and T stimulus, respectively. In **(B)**, T1 and T2 denote the first and second T stimulus in TT sequence, respectively; and the green and blue solid lines denote the waveforms evoked by the T1 and T2 stimulus, respectively.

Similar results could be found from both the TFDs (Figure 2) and scalp P300 ERP distributions (Figure 3). In Figure 2, when subjects responded to the presented T stimulus, the brain activity in low frequency range (i.e., range of 1–10 Hz) could be found in both ST (Figure 2A) and TT (Figure 2B) sequences on electrode Pz, respectively.

As illustrated in both Figures 2, 3, the obvious difference between two sequences was that, even though the TFDs in low frequency range could be found in TT sequence when both T stimuli were presented, the attenuated TFDs (Figure 2B) and P300 amplitudes (Figure 3B) were demonstrated for the second T stimulus (T2).

**Figure 4:**
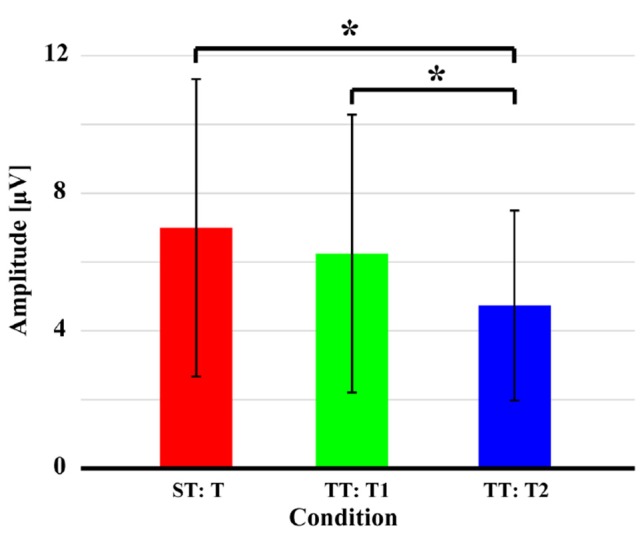
P300 amplitudes evoked by the T in both ST and TT sequences. The black solid asterisks denote the significant (*p* < 0.05) differences of P300 amplitudes between two conditions. Error bars denote the standard deviations of P300 amplitudes among 22 subjects. Values are the means and standard deviations (Mean ± SD) of P300 amplitudes.

Figure 4 displays the quantitative differences of P300 amplitudes between two sequences. As demonstrated, no significant difference (*t* = 1.628, *p* = 0.059, *df* = 21) of P300 amplitude between T in ST and T1 in TT was found; while significantly larger amplitude evoked by T in ST than that evoked by T2 in TT (*t* = 3.375, *p* = 0.001, *df* = 21) and larger amplitude evoked by T1 than that evoked by T2 in TT (*t* = 2.963, *p* = 0.004, *df* = 21) were found. For latency, longer latency for T2 was demonstrated, compared to that of T1 in TT (*t* = −1.467, *p* = 0.079, *df* = 21) and T in ST (*t* = −0.801, *p* = 0.216, *df* = 21), although these were insignificant. Meanwhile, no difference of latency (*t* = 0.271, *p* = 0.605, *df* = 21) between T in ST and T1 in TT was found.

**Figure 5:**
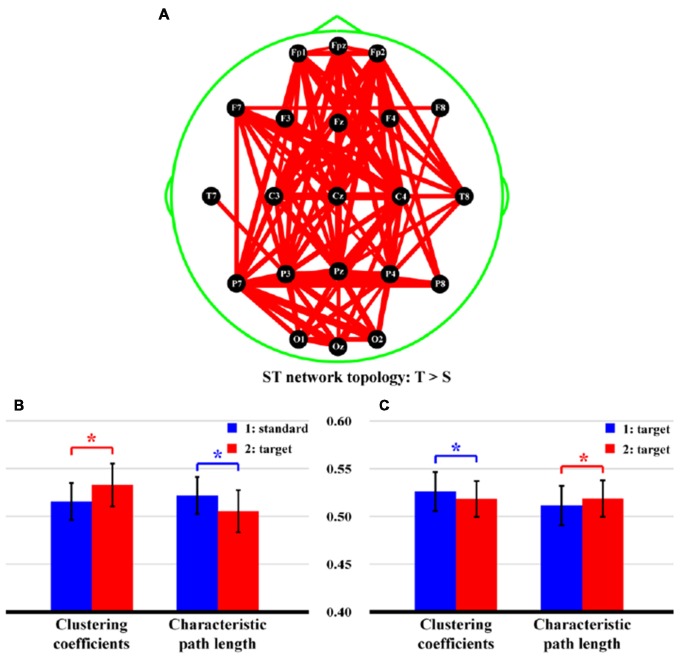
Differences of network topologies and properties between two stimuli in both ST and TT sequences. **(A)** Network topology of ST sequence. **(B,C)** Network properties of ST and TT sequences, respectively. In **(A)**, the red lines denote the stronger network edges of T than that of S stimulus; and the line widths denote the quantitative differences of edge strengths between two stimuli. In **(B,C)** the blue and red bars denote the network properties of first and second stimulus, respectively. *Indicates *p* < 0.05. Error bars denote the standard deviations of network properties among 22 subjects. Values are the means and standard deviations (Mean ± SD) of network properties.

Figure 5 shows the statistical differences of network topologies (Figure 5A) and network properties (Figures 5B,C) between two stimuli for both ST and TT sequences. Particularly, in Figure 5A, stronger network edges connecting frontal and parietal lobes were demonstrated for ST sequence (*p* < 0.05, FDR correction), which was also evaluated by higher network efficiency (Figure 5B, CC: *t* = −4.289, *p* = 0.000, *df* = 21; CPL: *t* = 4.114, *p* = 0.000, *df* = 21), when a T stimulus was presented after an S. However, for TT sequence, only smaller CC (*t* = 3.201, *p* = 0.002, *df* = 21) and longer CPL (*t* = −3.142, *p* = 0.003, *df* = 21), which reflected the attenuated network efficiency (Figure 5C), were demonstrated when an identical T stimulus was presented after the last T.

**Figure 6:**
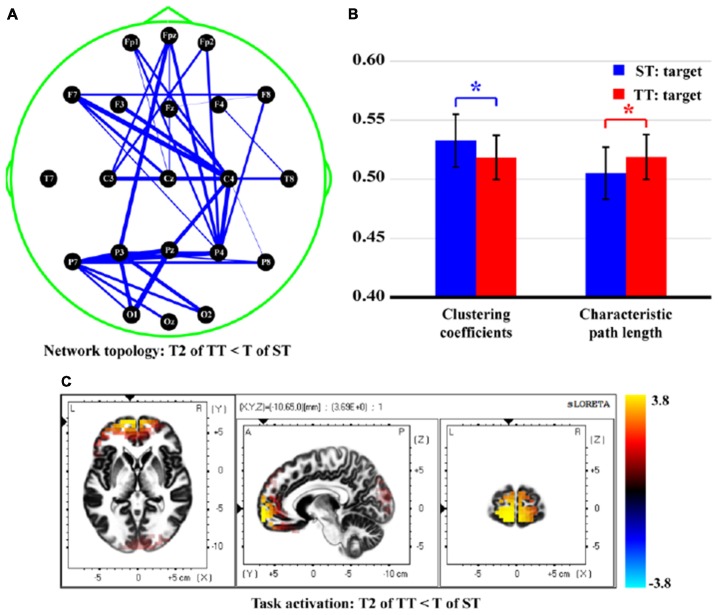
Differences of network topology, properties, and task activation between T in ST and T2 in TT. **(A)** Network topology. **(B)** Network properties. **(C)** Task activation. In **(A)**, the blue solid lines denote the weaker network edges of T2 in TT compared to that of T in ST; the line widths denote the quantitative differences of edge strengths between two conditions. In **(B)**, the red and blue bars denote the network properties corresponding to T2 in TT and T in ST, respectively. *Indicates *p* < 0.05. Error bars denote the standard deviations of network properties among 22 subjects. Values are the means and standard deviations (Mean ± SD) of network properties.

Figure 6 finally demonstrates the differences of network topology, properties, and task activations between T in ST and T2 in TT. Attenuated network edges (Figure 6A, *p* < 0.05, FDR correction) and properties [smaller CC (*t* = 3.573, *p* = 0.000, *df* = 21) and longer CPL (*t* = −3.532, *p* = 0.001, *df* = 21), Figure 6B] were found in TT sequence. Meanwhile, Figure 6C illustrates the stronger (*p* < 0.05, FDR correction) task activity mainly in frontal, temporal, and occipital lobes, when a T stimulus was presented after an S. Concretely, brain regions in both hemispheres, which included middle frontal gyrus (BA10, 11), inferior frontal gyrus (BA10, 11), superior frontal gyrus (BA11), middle temporal gyrus (BA21, 38), superior temporal gyrus (BA38), middle occipital gyrus (BA18), and cuneus (BA18, 19), were found to be significantly different between two sequences.

## Discussion

In this study, subjects were requested to mentally count the number of T stimulus as quickly and correctly as possible, once they noticed the presentation of a T stimulus. In the brain, target information was processed in multiple procedures including stimuli perception, information integration, decision processing, and neuronal response, which was thought to be attributed to the interacted activity in the brain (Zhang et al., 2014; Li et al., 2015, 2016).

The sequence effect originated in part from the operation of immediate memory for the preceding stimulus train (Polich and Bondurant, 1997). As sequence unfolded, variation in P300 amplitude was induced due to an automatic concomitant shift in perceptual expectancy fostered by stimulus changes. The P300 indexed an updating process, a T stimulus after long interval produced great template updating to refresh the representation (Polich, 1990; Gonsalvez et al., 2007) and, thus, produced large P300 amplitude. The TFDs in Figure 2 and scalp P300 ERP distributions in Figure 3 showed the effect of stimulus sequence on P300. Although T stimulus could evoke a P300 in both ST and TT sequences, the context (i.e., S or T) preceding an adjacency T was demonstrated to affect the generation of P300. Particularly, ST sequence evoked a larger P300 amplitude than TT sequence, which was also illustrated by the difference of P300 amplitude between different conditions in Figure 4.

Since no response was required for the S in ST sequence, brain resources were then allocated to react to the T. Therefore, the T stimulus experienced a stronger network topology (Figure 5A) that connected multiple regions including frontal and parietal lobes, compared to the S one. In essence, network properties can detect functional integration and segregation of a given network (Rubinov and Sporns, 2010), and the larger CC and shorter CPL denote an increase of information processing efficiency during task. In this study, the larger CC and shorter CPL of ST sequence shown in Figure 5B thus quantitatively measured the increased efficiency when processing T-related information. However, if two identical T stimuli were presented, the target-target perception reminded the brain to respond equally to both stimuli. In essence, a relatively long TTI (e.g., 6 s or longer) could eliminate the effect of target probability (also stimulus sequence), and the P300 generation system could then recover fully from the last use (Polich, 1990; Polich and Bondurant, 1997). In our present study, the TT sequence produced a relatively short TTI of 2.25 s. Although both P300 ERPs were evoked in TT sequence, due to short TTI, related brain resources could not fully and efficiently respond to the T2, which thus resulted in decreased P300 amplitude, prolonged P300 latency, and suppressed network properties (Figure 5C).

Once perceiving target information in the brain, related regions would be activated to be responsible for the corresponding dynamic processes, which included encoding, updating, and decaying of immediate memory trace (template) of the T stimulus (Owen et al., 1996; Polich and Criado, 2006; Luck et al., 2010). In this study, target information was first perceived and integrated in occipital lobe. Frontal lobe is demonstrated to be involved in multiple cognitive tasks and associated with effort and task difficulty (Paus et al., 1998). Here, the activation in frontal may reflect the general role of frontal lobe in response preparation and the monitoring of task information (Carter et al., 1998), which might relate to the early P300 process (i.e., command coding and decision processing; Li et al., 2009). Afterwards, the memory template of T stimulus was updated and transmitted to parietal lobe, a clear P300 component with large amplitude was thus evoked. As illustrated, the short interval affected the brain response to the T2 in TT sequence; we thus observed the stronger activations in multiple brain regions, such as middle frontal gyrus, superior frontal gyrus, and cuneus in Figure 6C for ST rather than TT sequence.

Findings of our study demonstrated the effect of stimulus sequence on brain network that subserved the target information processing in the brain. In the oddball task, ST sequence experienced an efficient allocation of brain resources, along with the high network efficiency, which would then promote the generation of P300 component with large amplitude. One possible limitation of this study was that subjects were all males, the gender difference might influence the findings to some degree, which was needed to be considered in the future work. Meanwhile, the investigation of single trial amplitude might be also helpful to deepen our understanding of this issue; in our future study, the combined single trial amplitude and brain network analysis could be considered, as well.

## Author Contributions

FL and PX conceived and designed the experiments and wrote the manuscript. FL performed the experiments. FL, CY, YJ, and YL analyzed the dataset. YS, JD, DY, and YZ provided some useful suggestions in manuscript writing.

## Conflict of Interest Statement

The authors declare that the research was conducted in the absence of any commercial or financial relationships that could be construed as a potential conflict of interest.

## Footnotes

^1^http://www.nitrc.org/projects/bct/
